# Advanced DNA-Based Point-of-Care Diagnostic Methods for Plant Diseases Detection

**DOI:** 10.3389/fpls.2017.02016

**Published:** 2017-12-06

**Authors:** Han Yih Lau, Jose R. Botella

**Affiliations:** ^1^Biotechnology and Nanotechnology Research Centre, Malaysian Agricultural Research and Development Institute, Serdang, Malaysia; ^2^Plant Genetic Engineering Laboratory, School of Agriculture and Food Sciences, University of Queensland, Brisbane, QLD, Australia

**Keywords:** point-of-care diagnostic, isothermal amplification, LAMP, HDA, RCA, RPA

## Abstract

Diagnostic technologies for the detection of plant pathogens with point-of-care capability and high multiplexing ability are an essential tool in the fight to reduce the large agricultural production losses caused by plant diseases. The main desirable characteristics for such diagnostic assays are high specificity, sensitivity, reproducibility, quickness, cost efficiency and high-throughput multiplex detection capability. This article describes and discusses various DNA-based point-of care diagnostic methods for applications in plant disease detection. Polymerase chain reaction (PCR) is the most common DNA amplification technology used for detecting various plant and animal pathogens. However, subsequent to PCR based assays, several types of nucleic acid amplification technologies have been developed to achieve higher sensitivity, rapid detection as well as suitable for field applications such as loop-mediated isothermal amplification, helicase-dependent amplification, rolling circle amplification, recombinase polymerase amplification, and molecular inversion probe. The principle behind these technologies has been thoroughly discussed in several review papers; herein we emphasize the application of these technologies to detect plant pathogens by outlining the advantages and disadvantages of each technology in detail.

## Introduction

Agriculture has an estimated value of $1500 billion US dollars (USD) per year (Agrios, 2005). However, an important amount of agricultural product is lost every year due to multiple diseases, and this problem is especially acute developing countries (Agrios, 2005; Strange, 2012) making crop disease management a priority in agriculturally based economies. In cases were resistant varieties are not available, the best option is to detect the presence of pathogens in the field as early as possible and thus avoid the onset of the disease. Hence, the effectiveness of many integrated pest management strategies are highly dependent on the availability of fast, sensitive and specific diagnostic methods. It is therefore important to develop more efficient technologies to detect crop diseases and effectively link them to decision bodies in order to efficiently deploy the necessary responses and safeguard agricultural systems.

Many methods have been developed to identify plant pathogens (Punja et al., 2007). The earliest conventional methods used symptom observation, involving field inspections to identify disease symptoms as well as laboratory tests such as pathogen culture on selective media followed by physiological, biochemical and pathogenicity tests (Horsfall and Cowling, 1977). Although conventional methods are reliable, they are time consuming and require experienced plant pathologists to identify the pathogen responsible for the disease. These reasons made it desirable to develop detection methods with higher sensitivity, specificity and speed for plant pathogen identification.

Antibody technology has been used in plant diagnostics since the 1980s and many reviews on this technology have been published (Alvarez, 2004; Narayanasamy, 2011). Antibody-based diagnostic methods for plant pathogen detection have become popular and powerful tools because of their speed, sensitivity and inexpensive nature. However, polyclonal antibodies (PAbs) against plant pathogens produced by animal immunization may show cross-reactivity with unrelated pathogenic species due to their limited specificity (Macario and Conway de Macario, 1985). With the development of monoclonal antibodies (MAbs), specificity was improved since they target a single epitope in a pathogen protein (Thornton, 2009). Hence, various antibody-based diagnostic methods such as enzyme-linked immunosorbent assays (ELISAs) (Comstock, 1992; Kokoskova and Janse, 2009), immunoblot (Novakova et al., 2006), immunofluorescent test (Malin et al., 1983) and lateral flow devices (LFD) (Tomlinson et al., 2010d) have been developed and widely used to identify plant pathogens. However, MAbs are expensive to produce and it has been reported that closely related species may share common epitopes and cause MAbs to react positively (Teitelbaum et al., 1991; Gorris et al., 1994).

The advent of the polymerase chain reaction (PCR) in the 1980s enabled scientists to explore and develop DNA-based approaches for plant pathogen detection. As a result many PCR-based diagnostic methods for the identification of plant pathogens have been reported (Ward et al., 2004; Vincelli and Tisserat, 2008). Furthermore, the amplification of pathogen-specific sequences and the coupling of PCR with other techniques have been described in order to improve specificity and sensitivity (Merighi et al., 2000; Brasileiro et al., 2004; Carrasco-Ballesteros et al., 2007). For example highly specific immunocapture-PCR (IC-PCR) has been used for viral detection combining conventional PCR amplification with antibody-captured viral particles. This approach increased sensitivity by 250-fold compared to direct PCR amplification (Wetzel et al., 1992) enabling successful detection of the bacterial blight disease in Anthurium propagation material (*Xanthomonas axonopodis* pv. *dieffenbachiae*) (Khoodoo et al., 2005).

A combination of conventional PCR and enzyme-linked immunosorbent assay (ELISA) termed PCR-ELISA was developed in the early 1990s (Nutman et al., 1994). The assay involves hybridization of the labeled PCR product to an immobilized probe on an ELISA plate followed by addition of an enzyme conjugate and a substrate to analyze the captured PCR product using a photometric measurement. This assay enabled the detection of a single amplicon population among several others that were generated in a multiplex reaction (Laitinen et al., 2002). This technique has been successfully used to detect viruses (Shindo et al., 1994), bacteria (Daly et al., 2002) and fungi (Bailey et al., 2002; Somai et al., 2002) with higher specificity than conventional PCR. However, despite its high specificity, the assay generated false positive results while detecting *Neisseria meningitides* (Borrow et al., 1998) and *Mycobacterium tuberculosis* (Kent et al., 1995; Gillespie et al., 1997) as the PCR amplified DNA was found to hybridize with the ELISA probe from other species.

An important improvement in DNA-based diagnostic methods came with the development of real-time quantitative PCR which allowed not only to detect the presence of pathogens but also to quantify pathogen levels in tissue samples thus allowing to determine the severity of the pathogen infection (Heid et al., 1996). A drawback of this technology is the requirements of expensive equipment and reagents which limits its use as a rapid cost-effective diagnostic method. In addition, the high sensitivity of the assay increases the risk of detecting even small amounts of contamination in the reagents or biological samples resulting in the diagnosis of false positives creating the need for normalization steps or pre-read runs to guarantee accuracy of results (Wong and Medrano, 2005; Nowrouzian et al., 2009).

Although PCR based assays have improved the sensitivity and have been used for multiplex pathogen detection (Price et al., 2010; Araujo et al., 2012; Dai et al., 2013), the assays are still prone to non-specific DNA amplification resulting in false positive results while performing multiplex detection on unknown pathogens in diseased plants tissues (Markoulatos et al., 2002; Sint et al., 2012).

## Point-Of-Care Testing for Plant Pathogens

Point-of-care (POC) diagnostic assays which do not require sophisticated equipment and can be rapidly and cheaply performed in the field are in high demand (Yager et al., 2006). PCR based methods have multiple advantages over other technologies but require an electricity supply to perform the temperature changes required for DNA amplification; seriously limiting its adequacy for POC applications (Barany, 1991). As an alternative, isothermal DNA amplification methods are ideally suited to overcome this limitation (Gill and Ghaemi, 2008; Craw and Balachandran, 2012). For instance, isothermal amplification combined with lateral flow strips and portable fluorometers has been successfully used for POC detection of pathogen DNA (Notomi et al., 2000; Vincent et al., 2004; Piepenburg et al., 2006; Chow et al., 2008; Lutz et al., 2010; Mahalanabis et al., 2010; Rohrman and Richards-Kortum, 2012). Nevertheless, portable fluorometers are expensive and not necessarily suited for use in the field exposed to adverse weather conditions, thus limiting their widespread adoption. A POC diagnostic assay technology integrating the entire process from sample preparation to visualization of results is still elusive. Agricultural industries could greatly benefit from the availability of convenient and cheap POC assays as crop field locations can be far away from analytical laboratory setups and sample transport can pose logistical problems.

## Point-Of-Care DNA Extraction Methods

An effective POC DNA extraction method is essential to develop rapid and user friendly molecular diagnostic assays but *in-field* sampling is rarely discussed when describing DNA-based diagnostic methods. This non-trivial task to consistently generate a fixed DNA input of good quality (i.e., absent of potential inhibitors) has repercussions on any assays’ performance. When dealing with plant tissues, the DNA extraction method requires the ability to efficiently remove a number of chemicals that can inhibit the DNA amplification reaction (Ikeda et al., 2008).

A LFD DNA extraction method has been reported as rapid and efficient for POC testing and has been successfully used in plant pathogen detection (Tomlinson et al., 2010a,b). This method involves sample disruption in extraction buffer using metal ball bearings before transferring the lysate onto the release pad of a LFD nitrocellulose membrane. A small piece of membrane is then excised and added into the DNA amplification reaction such as PCR or other isothermal amplification methods. The isolated DNA is very stable on the membrane at room temperature which allows the extraction to be performed in the field (Tomlinson et al., 2010a).

Solid Phase Reversible Immobilization (SPRI) is another DNA extraction method with potential POC application (Wee et al., 2015). A low cost DNA/RNA purification process using common filter pipette tips in conjunction with SPRI technology (Deangelis et al., 1995) to consistently extract DNA/RNA to a precise concentration that can be used immediately for downstream isothermal amplification has been recently reported (Wee et al., 2015). Magnetic SPRI bead-based extraction is ideal for POC applications because the only equipment required are a magnet and a micropipette. After macerating a single leaf disk with a plastic pestle in lysis buffer, the plant lysate was cleared of cellular debris by passing the lysate through a filtered pipette tip. DNA was then purified from the lysate using SPRI and the extracted DNA was directly used in the amplification reaction. This DNA extraction method has been used on various diagnostic applications including human and plant diseases (Ng et al., 2015; Wee et al., 2015).

A recent report has described the development of an extremely simple cellulose-based dipstick that allows plant sample processing in as little as 30 s (Zou et al., 2017). Plant tissues are macerated by shaking in a tube containing extraction buffer and 1–2 ball bearings for 8–10 s. A cellulose dipstick is inserted in the tube containing the sample before washing it three times in a second tube containing wash buffer and finally into the tube containing the amplification mix. The technology works efficiently in multiple cultivated species including rice, wheat tomato and sorghum as well as notoriously difficult tissues such as leaves from mature trees (mandarin, lime, and lemon). It can be used to detect pathogen DNA as well as RNA from infected tissues and works with multiple amplification methods such as PCR, LAMP, and RPA. Direct comparison studies have shown that this technology is as sensitive as SPRI but is cheaper, faster and does not require any pipetting steps.

## Application of Nucleic Acid Isothermal Amplification Techniques in Plant Disease Detection

Enzyme mediated *in vitro* amplification of nucleic acids has become an essential tool in molecular biology since 1980’s (White et al., 1989). PCR is one of the most widely used methods for the detection and identification of pathogens by targeting specific sequences in their genomic DNA. Although PCR is highly sensitive and robust, it is constrained by a number of technical limitations. For instance, specificity is highly dependent on the primers used and its inherent sensitivity makes it prone to false positives due to sample cross-contamination. Besides, PCR also requires electrically powered equipment to perform the thermal cycling which limits its use for point-of-care diagnostics. A number of alternative isothermal techniques are now available that can obviate the need for a thermal cycler although each has strengths and weaknesses.

### LAMP

**FIGURE 1:**
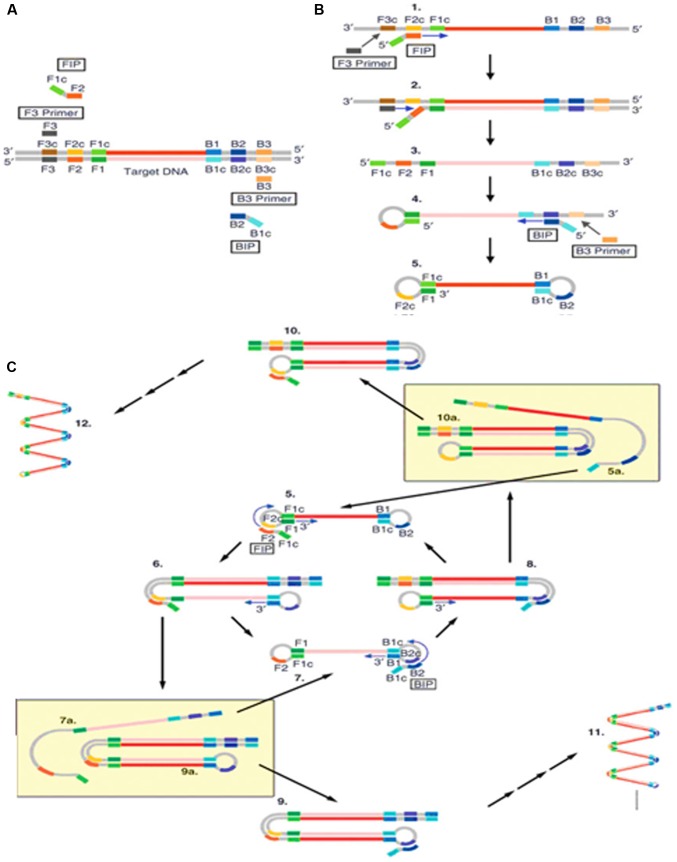
Schematic outline of loop-mediated isothermal amplification (LAMP) (Tomita et al., 2008). **(A)** LAMP involves two sets of primers to target six distinct regions. **(B)** The inner primer containing sense and antisense sequences of the target DNA hybridizes to the targeted sequence and initiates DNA synthesis. The outer primer carries out the strand-displacement DNA synthesis and produces a single stranded DNA which works as a template for second inner and outer primers for DNA synthesis that hybridize to the other end of the target to form a DNA loop structure. **(C)** From the double stem-loop structure, the inner primer binds to the loop and synthesizes a new strand. The extension of the primer opens the loop at the 5′ end and again the outer primer strand displaces the newly form longer DNA to produce ssDNA to form a DNA loop. LAMP produces loop structure DNA in various sizes.

Loop-mediated isothermal amplification (LAMP) is a nucleic acid amplification method developed in Notomi et al. (2000). It has been widely used due to its high efficiency, specificity, simplicity and quickness. LAMP requires two long outer primers and two short inner primers that recognize six specific sequences in the target DNA. The first inner primer containing sense and antisense sequences in the DNA will hybridize the target sequence and initiate DNA synthesis (**Figure 1**; Tomita et al., 2008). Next, the outer primer carries out the strand-displacement DNA synthesis and produces a single stranded DNA which works as a template for the second inner and outer primers producing a DNA molecule with a loop structure. The unremitting cycling reaction accumulates products with repeated sequences of target DNA of different sizes.

Loop-mediated isothermal amplification has three major advantages. Firstly, it can be carried out at a constant temperature with a short reaction time. This rapid isothermal process makes it ideal for point-of-care detection of plant pathogens in the field (Fukuta et al., 2014) and has been used to detect the plum pox virus in 2.5 h (Hadersdorfer et al., 2011). Secondly, it has very high amplification efficiency and sensitivity as it generates large amounts of PCR product with low amounts of input DNA (Tomlinson et al., 2010c; Bhat et al., 2013). Finally, this method is relatively cost effective as it requires simple equipment to perform the assay. Furthermore, there have been reports stating that LAMP generates amplicons with several inverted repeats which could be potentially used to increase the sensitivity in hybridization assays, such as LAMP-ELISA hybridization (Lee et al., 2014) and LAMP incorporated with colorimetric gold nanoparticle hybridization probes (Watthanapanpituck et al., 2014).

Although LAMP is an ideal isothermal method for in field POC plant pathogen detection, it does possess certain limitations. Firstly, the design of the LAMP primers is complicated and non-intuitive, making it difficult for non-specialists. Even though there is available software for LAMP primer design, optimal primers performance is not guaranteed as it is usually the case for PCR. In addition, LAMP amplicons contain a mixture of stem-loop DNA molecules of different sizes which are not suitable for gene cloning purposes or to identify specific targets based on size differences. However, the size limitation was overcome by Nagamine et al. (2002) who developed a modified LAMP method that generates uniform and single stranded DNA amplicons through *Tsp*RI digestion before extending the products using a primer to produce strand-specific DNA fragments.

Since LAMP has potential for POC diagnostic purposes, several simple read-out methods have been combined with LAMP to replace the traditional gel electrophoresis analysis in order to detect the presence of amplicons. The simplest and cheapest methods use metal-ion indicators such as hydroxynaphthol blue (HNB) or SYBR green as DNA dyes to visualize the final products (Chen et al., 2012; Duan et al., 2014). By adding color indicators into the LAMP reaction prior to amplification, the products can be detected by the naked eye using a simple colorimetric assay. Using this read-out method, it was possible to detect as low as 10 copies of target DNA (Chen et al., 2012). The colorimetric readout has been successfully used for naked-eye detection of potato leafroll virus and *Fusarium oxysporum* (Ahmadi et al., 2013; Zhang et al., 2013). Nevertheless, in our experience the color changes induced by the above-mentioned indicators can be quite subtle and, even though they might be possible to observe in a laboratory environment, they are difficult to detect in the field due to the different light conditions at different times of the day.

Alternatively, LAMP has been combined with ELISA by incorporating antigen-labeled nucleotides into the LAMP amplicons during the amplification process. The generated amplicons are then hybridized to specific immobilized oligonucleotide probes which are later analyzed by immunoassay. The main advantage of LAMP-ELISA is its ability to process hundreds of samples simultaneously within a few hours (Ravan and Yazdanparast, 2012; Lee et al., 2014). The combination of LAMP with ELISA provides very high sensitivity with a single copy of target DNA being successfully detected (Lee et al., 2014). However, this technique requires skilled labor as it involves complicated ELISA steps.

Following the use of optical and colorimetric readout systems, electrochemical sensors capable of detecting signal changes caused by the electron transfer in double stranded DNA have been used to detect the presence of amplified DNA (Ferguson et al., 2009). The integration of LAMP with the electrochemical sensor offered a robust platform for pathogen detection as it was highly sensitive, detecting as low as 10 copies of pathogen genomic DNA (Hsieh et al., 2012). The applications of LAMP-biosensor technology are increasing in all fields, from clinical molecular diagnostics to environmental monitoring, but its application is still fairly new in plant pathogen detection. Therefore, LAMP-biosensor technology has a strong potential for in field testing, detection and identification of plant diseases.

### HDA

**FIGURE 2:**
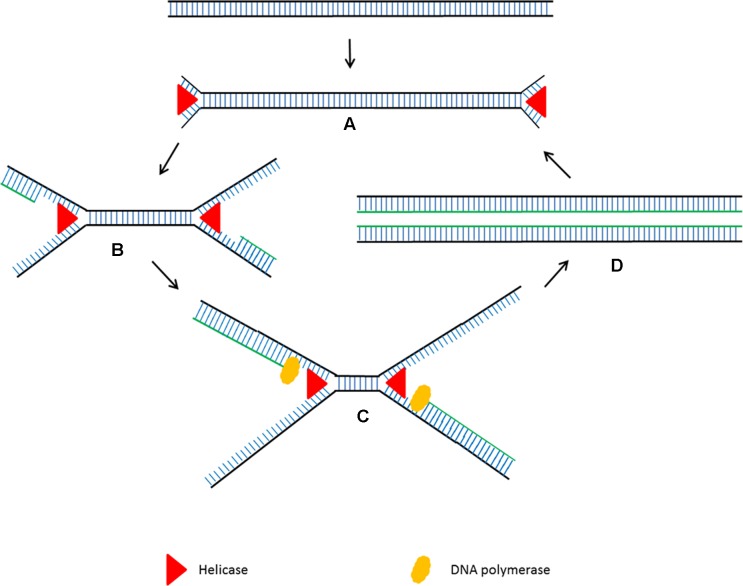
Schematic outline of helicase dependent amplification (HDA). **(A)** Helicase opens the dsDNA. **(B)** The primers anneal to the target sequences. **(C)** Primer extension by DNA polymerase. The newly formed dsDNAs are opened by helicase and the process starts again. **(D)** The newly formed dsDNAs are opened by helicase and the process starts again.

Helicase dependent amplification (HDA) is an alternative isothermal technique developed by New England Biolabs in 2004 (Vincent et al., 2004). This isothermal technique is very similar to the standard PCR but it does not require heat denaturation to separate the double stranded DNA and allows primers to anneal to its complementary target sequences. HDA uses DNA helicase to generate single stranded DNA for primer annealing followed by primer extension at isothermal conditions (**Figure 2**). Single stranded binding protein (SSB) and MutL endonuclease are added to the reaction to prevent re-hybridization of complementary ssDNA strands to reform the dsDNA. Detectable amounts of PCR amplicons for downstream analysis are generally generated within 60 min by the HDA method (Vincent et al., 2004).

Helicase dependent amplification has become a popular isothermal technique due to its simple reaction steps. Although it amplifies the target sequences using a pair of primers using the same principle as PCR, the steps are much simpler as it does not need multiple temperature cycling steps. Although LAMP and had are both isothermal, LAMP requires four intricately designed long primers which need an initial heat denaturation step before amplification at a lower temperature (Nagamine et al., 2002). HDA has been successfully used to detect pathogen genomic DNA present in a crude mixture of human blood sample with high sensitivity (Vincent et al., 2004). Although HDA’ s characteristics seem perfect for the development of simple point of care diagnostic assays, the main drawback is that it requires complex optimization to ensure a coordinated enzyme activity between the helicase and DNA polymerase.

Additionally, the presence of SSB and MutL, which are essential to prevent ssDNA from re-hybridizing to form dsDNA can potentially affect the final results significantly (Vincent et al., 2004). Furthermore, there are some reports stating that HDA is inefficient when amplifying long targets (Guatelli et al., 1990), probably due to the fact that the UvrD helicase has limited unwinding speed (20 bp/s) and process less than 100 bp per binding (Ail et al., 1999). MutL is able to enhance the UvrD unwinding activity but does not increase the processing rate (Mechanic et al., 2000). A significant improvement has been made with the discovery of a thermostable UvrD helicase (Tte-UvrD) from *Thermoanaerobacter tengcongensis* suitable for amplification at a higher temperature (An et al., 2005). The use of Tte-UvrD allows HDA to be performed at a higher temperature, decreasing the re-annealing of single stranded DNA and therefore obviating the need for SSB and MutL.

Currently, HDA is commonly used in human clinical applications such as the diagnosis of *Salmonella paratyphi* (Faizul Rahman et al., 2014) and diarrhea-causing pathogens (Huang et al., 2013; Eckert et al., 2014), as well as veterinary applications such as detection of *Streptococcus equi* causing strangles in horses (Artiushin et al., 2011). However, its application to plant pathogen detection is still limited and has only been used to identify citrus leprosis virus C and tobacco mosaic virus (Corona et al., 2010). In order to improve sensitivity, HDA has been combined with other technologies such as ELISA (Gill et al., 2007) and gold nanoparticles (Gill et al., 2008) to detect *Helicobacter pylori*. The results of both HDA-ELISA and HDA-nanoparticles showed a 90% increase in sensitivity and specificity compared to the original HDA assay.

### RCA

**FIGURE 3:**
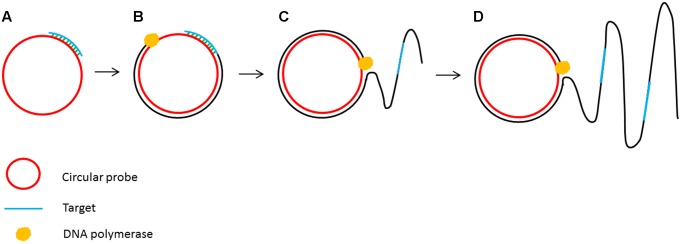
Schematic outline of rolling circle amplification (RCA). **(A)** A primer complementary to a region of a circular probe anneals to the circular template. **(B)** DNA polymerase initiates the DNA synthesis. **(C)** Strand displacement allows the continuation of DNA synthesis along the circular template. **(D)** DNA synthesis continues to generate a long ssDNA product.

The principle of isothermal amplification has been also used to amplify circular DNA in a process known as rolling circle amplification (RCA) (Fire and Xu, 1995). RCA involves using a DNA polymerase with strand displacement activity (such as ϕ29 DNA polymerase) to extend a single primer annealed to a circular DNA template. The strand displacement activity allows the newly synthesized DNA to displace the previously generated DNA releasing ssDNA (Blanco et al., 1989). This enzymatic process of primer extension combined with strand displacement generates a long single stranded DNA containing a repeated sequence complementary to the circular template (**Figure 3**).

**FIGURE 4:**
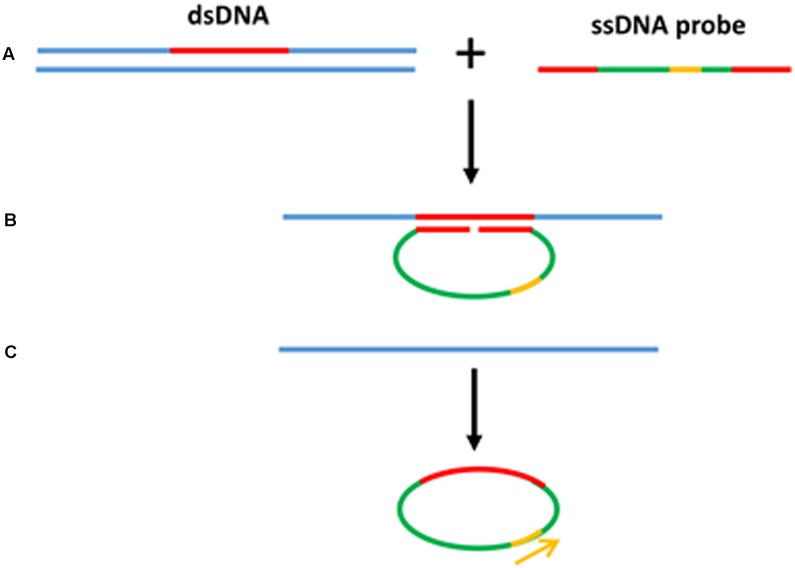
Padlock probe assay with RCA. **(A)** Linear ssDNA probes contain two binding sites at both 3′ and 5′ ends to target the specific sequences. **(B)** Denaturation of dsDNA target sequence and hybridization of ssDNA probe toward the target region forming a loop. **(C)** After the ligation to form a circular probe, RCA primer binds to the primer target region and starts the RCA.

Rolling circle amplification offers the simplest available isothermal reaction mechanism. With additional manipulation, linear DNA is also suitable as a template for the RCA reaction. A linear ssDNA probe can be designed in such a way that it can be initially hybridized to the target sequence forming a loop and ligated to form a circular probe prior to performing RCA (**Figure 4**). This process, termed padlock probe assay, has been used in the detection of many plant diseases (Szemes et al., 2005; Slawiak et al., 2013; Tian et al., 2014). The high multiplexing potential and specificity of padlock probes followed by RCA also contributes to its popularity in multiplex detection of plant pathogens. In addition, RCA has been reported to have higher specificity and be less prone to non-specific amplification than PCR. Another advantage of the RCA method is that it allows to generate up to 0.5 megabases of DNA per probe in an overnight incubation (Baner et al., 1998) and generates 10^9^ or more copies of each circle in 90 min (Lizardi et al., 1998). Generating multiple copies of repetitive sequences offer an advantage in hybridization based readouts where the repetitive sequences can be easily captured to increase the sensitivity (Gusev et al., 2001; Nallur et al., 2001; Russell et al., 2014). Furthermore, RCA is resistant, or at least less prone to carry-over contamination of the amplification products because there is no new 3′-end ssDNA product generated throughout the RCA process, which could be a potential primer for non-specific amplification (Kobori and Takahashi, 2014).

Rolling circle amplification has been widely used for plant pathogen detection since early 2000s. Several techniques have been used in combination with RCA such as restriction fragment length polymorphism (RFLP) and direct sequencing to identify and classify plant pathogen efficiently with significantly lower effort and cost than conventional technologies (Schubert et al., 2007). Visualization of RCA products using naked eye by adding fluorescent dye has been used to detect more than 40 strains of *Fusarium* spp. (Davari et al., 2012). Ligation of padlock probes followed by RCA has also been developed for identification of fungal pathogens (Najafzadeh et al., 2011). Incorporating RCA with a variety of readout technologies such as microarrays, DNA biosensors and immune assays has significantly improved the sensitivity when compared to gel electrophoresis (Gusev et al., 2001; Nallur et al., 2001; Li et al., 2009; Ji et al., 2012; Russell et al., 2014). Although these read out methods seem as an ideal alternative for RCA based assays, they are expensive and involve complicated steps compared to simple monitoring a color change using naked eye.

### RPA

**FIGURE 5:**
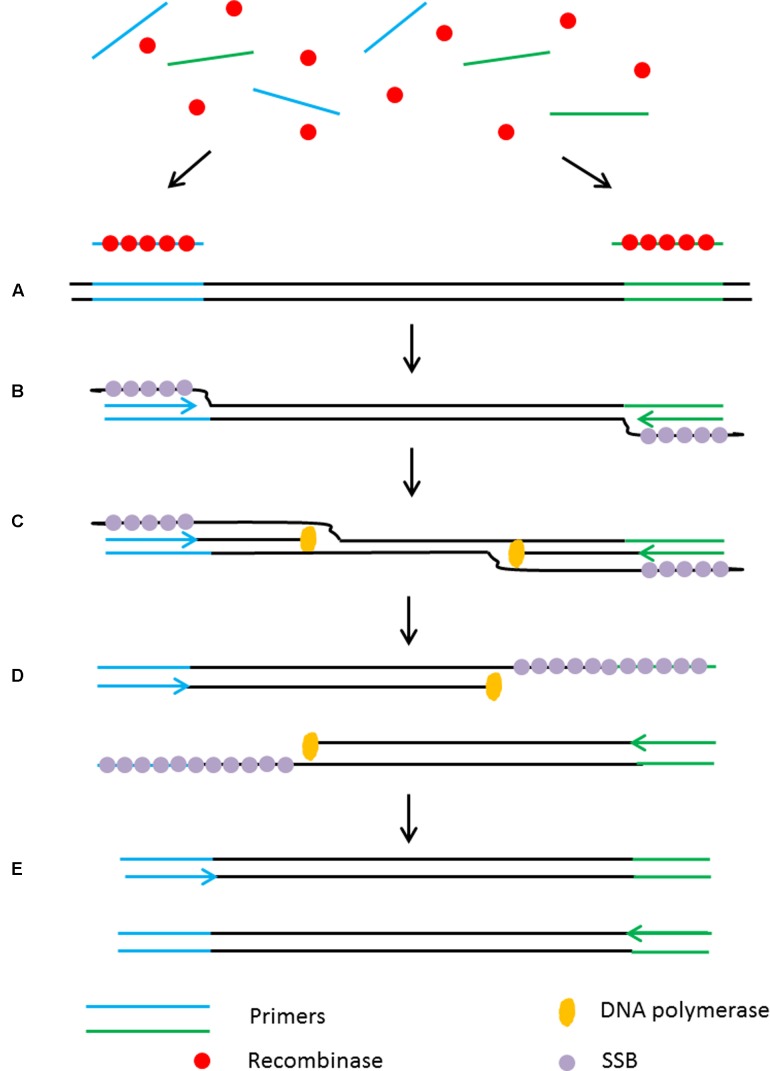
Schematic outline of the recombinase polymerase amplification (RPA). **(A)** Recombinase integrates with primers to form recombinase-primer complexes and target specific DNA sequences. **(B)** Strand exchange occurs and single stranded binding proteins (SSB) bind to the DNA to form a D-loop. **(C)** DNA polymerase initiates DNA amplification. **(D)** Displaced D-loop stabilized by SSB as amplification continues. **(E)** Two dsDNA molecules form and the entire cycle start again.

Recombinase polymerase amplification (RPA) is another isothermal technique that, like HDA, does not require an initial heating step to denature the target DNA (Piepenburg et al., 2006) as it relies on an enzymatic activity to separate the dsDNA in order to assist primer binding to the target sequences. The reaction begins with the integration of a recombinase protein with the primers prior to their annealing to specific sequences in the target. Following primer annealing, the recombinase dissociates from the primers and leaves their 3′ end accessible to the DNA polymerase to initiate the amplification. This creates a d-loop which is stabilized by a single stranded binding protein (SSB) to keep the DNA open as a DNA polymerase with strand displacement activity continues the amplification (**Figure 5**). Using RPA, billions of DNA copies can be generated efficiently in 60 min with an incubation temperature between 37°C and 42°C (Piepenburg et al., 2006).

The low incubation temperature and short reaction time (15–30 min) make RPA a suitable assay for use in point-of-care diagnostic applications. Furthermore, primer design is simple without consideration of annealing temperature as they form a complex with the recombinase to target the homologous sequences. RPA is highly sensitive with a detection limit as low as 6.25 fg of genomic DNA input with a specificity >95% (Boyle D.S. et al., 2014).

However, as with the other discussed technologies, RPA has some drawbacks as it can only amplify small DNA fragments (<500 bp), therefore it is not suitable in cases where amplification of full length genes is required. In addition, the longer primers (30–35 nt) required for RPA are prone to produce non-specific amplification at low temperature. Furthermore, the primers used in the RPA reaction frequently generate high background noise on negative and non-template control samples due to the formation of primer dimers thus affecting the sensitivity of the assay.

Many reports describing clinical RPA-based applications have been recently published (Boyle D.S. et al., 2014; Crannell et al., 2014; Kersting et al., 2014). In plants, a number of RPA-based applications have been described to detect plant pathogens with high sensitivity, specificity and cost effectiveness. A combination of reverse transcriptase-RPA (RT-RPA) and lateral flow has successfully detected little cherry virus from crude extracts being more cost effective than RT-PCR and more suitable for resource limited settings (Mekuria et al., 2014). RT-RPA has also been used to detect plum pox virus with higher sensitivity than conventional ELISA and immunostrip (Zhang et al., 2014). RPA-ELISA has been developed for sensitive, specific and cost-effective identification of plant pathogens (Santiago-Felipe et al., 2014). A naked eye assay which couples RPA with bridging flocculation of magnetic beads has been recently developed for efficient POC detection of plant and human pathogens (Ng et al., 2015; Wee et al., 2015). RPA followed by DNA electrochemical sensor as a readout platform has been reported as a new technology for plant pathogen detection (Lau et al., 2017). This RPA/electrochemical sensor is 10,000 times more sensitive than conventional polymerase chain reaction (PCR)/gel electrophoresis and it has successfully identified *Pseudomonas syringae* infected plant samples at an early infection stage before any symptoms are visible. Given that RPA is a fairly new isothermal technique, there is still more exploration to be done for point-of-care detection of plant pathogens.

## Multiplex Detection of Plant Pathogens

Development of technologies with multiplex detection capability is another challenge in plant disease diagnostics as they are more cost effective, reduce assay time and require minimal amount of sample. High throughput multiplex detection has been successfully achieved using real-time PCR on an OpenArray^TM^ platform (van Doorn et al., 2007), microsphere immunoassay technology (Charlermroj et al., 2013) and microarrays (Szemes et al., 2005). However, simpler but efficient multiplex detection methods capable to identify multiple pathogens simultaneously are still needed.

**FIGURE 6:**
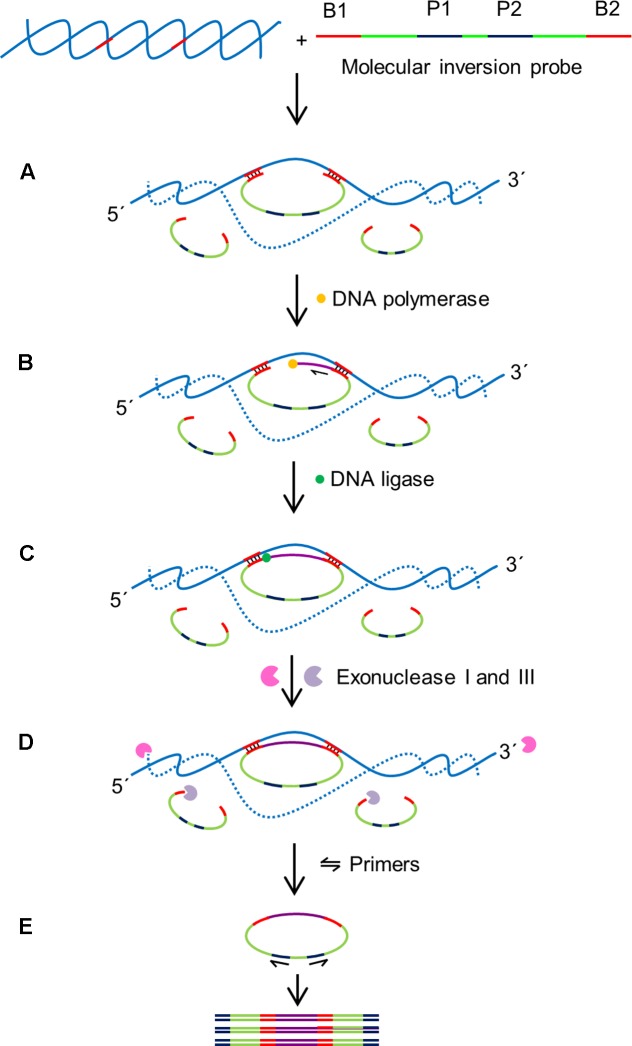
Schematic outline of MIP assay (Lau et al., 2014). MIP consists of two binding sites at 3′ and 5′ ends (B1 and B2) which are complementary to target sequences, and two universal primer sites (P1 and P2). B1 and B2 hybridize to specific sequences on the target with single stranded gap between two binding regions. **(A)** Hybridization: B1 and B2 bind to specific sequences on the target DNA creating single stranded gap between the binding domains of the MIP. **(B)** Gap filling: A DNA polymerase that lacks exonuclease and strand displacement activities synthesizes DNA from 3′ end of the MIP to 5′ end until the single stranded gap is filled. **(C)** Ligation: A DNA ligase ligates the 3′ end the 5′ end of the MIP creating a circular DNA. **(D)** Digestion: exonucleases I and III digest the linear MIPs and the DNA target in the reaction mixture leaving the circularized MIPs for amplification. **(E)** Polymerase chain reaction (PCR): A pair of universal primers (P1 and P2) amplifies the circularized MIP using the universal primer binding domains to generate PCR amplicons.

Molecular inversion probes (MIPs) enable cost effective multiplex diagnostics and has been often used in clinical applications. MIP is a long single stranded probe in excess of >100 nt long with two binding domains at the 5′ and 3′ ends (B1 and B2 in **Figure 6**). The binding domains are designed to be complementary to the target region of interest (**Figure 6**; Lau et al., 2014). This enables the MIP to form a circular loop with a single stranded gap in between the two binding regions. After annealing of the MIP, a DNA polymerase lacking 5′–3′ exonuclease and strand displacement activities initiates DNA synthesis from the 3′ end of the gap in a gap-fill reaction. DNA ligase is then used to ligate the newly synthesized strand and generate a circular DNA molecule. To ensure the sensitivity of the assay, an exonuclease digestion step is performed to remove the linear probes. The ligated MIPs are then amplified in a PCR reaction using a universal primer set targeted P1 and P2.

The highly multiplexable nature of MIP probes, which are able to distinguish thousands of targets in a single reaction is a major advantage of this assay. The binding domains at the 3′ and 5′ ends are connected by a DNA backbone; this design feature physically restricts the binding domains to a small region of the genome that needs to contain both recognition sequences, dramatically increasing the specificity of the MIP assays. In addition, the noise signal of the assay is decreased by enzymatically degrading the non-ligated linear MIPs by the exonuclease digestion with up to 99% efficiency. MIPs have been designed to detect up to 330 000 targets in a single reaction (Diep et al., 2012). The MIP multiplexing capability has been demonstrated in various clinical studies such as high-throughput analysis of single nucleotide polymorphisms, DNA methylation, detection of genomic copy number changes and other genotyping applications (Hardenbol et al., 2003; Wang et al., 2005; Diep et al., 2012). However, applications for plant diagnostics are still limited with the first report describing the successful detection of *Fusarium oxysporum* f.sp. *conglutinans* in infected *Arabidopsis thaliana* with a detection limit of 2.5 ng (Lau et al., 2014). In order to improve the efficiency of MIP assays, the MIPgen software has been developed for the design and performance prediction of individual MIPs (Boyle E.A. et al., 2014).

Serious limitations of these assays are the requirement for several temperature settings throughout the assay and a tedious yet complicated experimental process that makes it unsuitable for POC testing. Besides, the assay is time consuming taking in excess of 10 h to complete a reaction, making MIP assays only suitable for applications in the laboratory. MIPs with lengths of >100 bp require HPLC purification which increases the cost of oligonucleotide synthesis. Achieving a high level of multiplex detection involving thousands of MIPs in a single reaction requires a large initial investment. In addition, multiplex detection using MIP assays needs a complex read-out platform such as next generation sequencing, microarrays or biosensors (Lin et al., 2010; Wang et al., 2012; Carrascosa et al., 2014) further increasing the cost of the assay.

A recently published method using a combination of RPA and surface enhanced Raman scattering (SERS) has described the possibility of multiplex detection of plant pathogens in the field (Lau et al., 2016). Surface-enhanced Raman scattering (SERS) is a technique that provides enhanced Raman scattering patterns of the adsorbed molecules from metal nanoparticles surfaces upon a single laser excitation (Schlucker, 2014). SERS has been reported as a potentially powerful molecular spectroscopy detection tool (Moskovits, 2010), and a highly promising readout technology for rapid diagnostic assays (Anker et al., 2008; Bantz et al., 2011; Sharma et al., 2012). SERS produces narrow and distinct spectral peaks which provides higher precision after analysis compared to the standard fluorescent-based methods for highly multiplexed applications (Schlucker, 2009; Wang and Schlucker, 2013; Laing et al., 2016). Furthermore, SERS using different Raman reporters have enabled multiplex detection (Lai et al., 2015) especially in clinical applications (Maiti et al., 2011; Wang et al., 2015). The first report incorporating SERS with multiplex RPA for agricultural applications has appeared in 2016, detecting several plant pathogens (Lau et al., 2016). The technique has been successfully used to detect *Botrytis cinerea* in infected tomato plants in a field situation. Although this technique is ready for field application, it involves an expensive portable SERS reader which might limits its application.

## Discussion and Conclusion

The main benefit of POC diagnostic testing is to provide rapid results *in situ*, enabling farmers to make immediate management decisions and minimize crop loses. An additional advantage is the reduction in logistic problems associated with sample transportation to centralized laboratories for disease analysis and the concomitant labor costs. POC diagnostic kits should be portable and user friendly allowing a single operator with no specialized scientific skills to carry out the test. In this paper, some of the commonly used nucleic acid-based methods for plant pathogen detection were discussed. Although the vast majority of current applications are PCR-based, numerous research reports have established that the existing isothermal techniques can perform as well as or even better than PCR-based assays. The ability to perform reactions at a constant temperature makes isothermal techniques promising candidates for point-of-care diagnostic assays in low resource settings. Each of the isothermal and non-isothermal technologies discussed here has inherent advantages and limitations therefore the choice on the best technology to adopt will depend on the specific problem; i.e., the nature of the pathogen, the crop being monitored and the technological capability of the country.

Among the available isothermal techniques, LAMP and RCA are the best candidates for hybridization based applications because the repeated sequences present in the amplification products are able to increase detection sensitivity (Gusev et al., 2001; Nallur et al., 2001; Hsieh et al., 2012). However, both techniques produce isothermal amplification products in various sizes and are therefore not suitable for applications where a specific DNA fragment size is needed for identification. HDA and RPA isothermal technologies do not require a heat denaturation step and as a result can be performed directly at a constant temperature, a big advantage for field applications. Nevertheless, HDA and RPA are limited to targets shorter than 100 and 500 bp, respectively. Furthermore, despite the sensitivity and specificity of these isothermal techniques, they are not highly multiplexable, limiting their application to the detection of a single, or at best a limited number of pathogens in a single assay. To achieve the multiplex detection of plant pathogens, MIP is a promising method which can provide high specificity but its application in the field is quite limited due to its technical complexity. The development of RPA-SERS technology allows multiplex field detection to be performed within 90 min at a single constant low temperature (37°C) with high sensitivity and specificity, however, portable SERS readers are expensive.

Next generation sequencing (NGS) technologies have a huge potential in the diagnostic space as they can identify multiple pathogens in a single analysis without any previous knowledge of their nature (Massart et al., 2014; Withers et al., 2016; Rott et al., 2017). Nevertheless, they still need to overcome a number of limitations before they can be used for POC applications. NGS equipment is quite sophisticated, requiring a clean laboratory environment as well as a reliable power supply. Sample preparation for analysis is quite complex, requiring multiple steps, pipetting of small volumes, occasional use of centrifuges and final quality checks using bio-analyzing equipment. Finally, the current cost of NGS sequencing technologies, in the range of hundreds or thousands of dollars, is too high for POC routine diagnostic analyses although future developments might make them affordable for this type of applications.

In summary, traditional laboratory methods are accurate but labor intensive and slow and, most importantly, need highly specialized technical personnel. Unfortunately the availability of skilled plant pathologists is becoming a problem worldwide (Horsfall and Cowling, 1977). Antibody-based diagnostic methods are clearly faster than traditional techniques but specificity of detection can be sometimes strongly compromised due to cross reactivity resulting in erroneous pathogen identification (Franken et al., 1992). The limitations of antibody-based methods are further compounded by their short shelf life and batch to batch variability (Murphy et al., 2012). Nucleic acid-based methods provide higher specificity and have the potential to solve many of the problems experienced in antibody-based diagnostics (Ward et al., 2004; Vincelli and Tisserat, 2008; Hindson et al., 2011; Miotke et al., 2014). PCR is the most popular DNA-based identification technique for plant pathogens but it needs a power supply to achieve the continuous changes in temperature that are crucial for this technology to work, limiting its suitability for POC field applications (Markoulatos et al., 2002; Sint et al., 2012). Isothermal amplification systems can address this limitation and provide better sensitivity, specificity as well as capability in POC applications either by themselves or coupled with other technologies such as rapid readout methods to analyse the DNA amplification products.

## Author Contributions

All authors listed have made a substantial, direct and intellectual contribution to the work, and approved it for publication.

## Conflict of Interest Statement

The authors declare that the research was conducted in the absence of any commercial or financial relationships that could be construed as a potential conflict of interest.
